# Gut modulation to regulate NF-κB in colorectal and gastric cancer therapy and inflammation

**DOI:** 10.1007/s00262-025-04118-9

**Published:** 2025-07-12

**Authors:** Rowan Kearns

**Affiliations:** Ulster University, Life and Health Sciences, Coleraine, UK

**Keywords:** NF-κB pathway, Gut microbiota, Cancer, Inflammation, Dysbiosis

## Abstract

The nuclear factor-kappa B (NF-κB) pathway plays a pivotal role in cancer progression, immune regulation, and inflammation. Aberrant activation of this pathway, often driven by gut microbiota dysbiosis, contributes to tumorigenesis, therapy resistance, and chronic inflammation. Emerging evidence highlights the bidirectional interaction between gut microbiota and NF-κB signalling, suggesting that microbiota modulation may enhance cancer treatment efficacy and reduce treatment-induced inflammation. This review explores the mechanistic underpinnings of gut microbiota-mediated NF-κB regulation, focusing on microbial metabolites such as short-chain fatty acids (SCFAs) and microbial-associated molecular patterns, including lipopolysaccharides (LPS). It examines how conventional cancer treatments, chemotherapy, radiotherapy, and immune checkpoint inhibitors, exacerbate dysbiosis and NF-κB-driven inflammation, further complicating treatment outcomes. Additionally, this review evaluates the therapeutic potential of gut-targeted interventions, including probiotics, prebiotics, faecal microbiota transplantation (FMT), and dietary modifications, in restoring microbial homeostasis and modulating NF-κB signalling. Despite promising findings, challenges remain regarding the clinical translation of microbiota-based therapies, including the need for standardised microbiota profiling, regulatory frameworks, and long-term safety assessments. Advances in metagenomics and metabolomics are proposed as essential tools to personalise gut-targeted interventions and optimise cancer treatment strategies. Integrating gut modulation into oncology represents a paradigm shift, offering a holistic, patient-centric approach to cancer therapy. However, further research is required to validate these strategies and ensure their efficacy in clinical applications.

**Supplementary Information:**

The online version contains supplementary material available at 10.1007/s00262-025-04118-9.

## Introduction

Cancer remains a leading cause of mortality worldwide, with an estimated 19.3 million new cases and 10.0 million cancer deaths in 2020 alone [1]. This global burden is rising and projected to reach 28.4 million cases by 2040 (a − 47% increase from 2020) due to population growth and aging [2].

The nuclear factor-kappa B (NF-κB) pathway is a central regulator of cellular processes pivotal to inflammation, immune responses, apoptosis, and tumorigenesis [3]. Since its discovery by Sen and Baltimore in 1986 as a transcription factor involved in immunoglobulin κ-light chain gene expression in B cells, NF-κB has been associated in a wide array of physiological and pathological contexts [4]. The NF-κB pathway’s capacity to integrate diverse cellular signals establishes it as a fundamental regulator of both physiological and pathological processes [3]. While its physiological functions include maintaining immune surveillance and tissue homeostasis, aberrant activation is a hallmark of numerous cancers [5]. Dysregulated NF-κB promotes anti-apoptotic signalling, cellular proliferation, and angiogenesis, encouraging tumour initiation, progression, and metastasis [6, 7]. This pathway’s activation is particularly prevalent in inflammation-associated cancers, highlighting its role as both a driver of oncogenesis and a mediator of resistance to conventional cancer therapies, including chemotherapy and immunotherapy [8]. Importantly, NF-κB activation is also implicated in therapy resistance, it can inhibit chemotherapy or radiotherapy-induced apoptosis and foster tumour cell survival, as well as blunt the efficacy of immune-based therapies. Given its multifaceted role in cancer pathophysiology, NF-κB is recognised as a promising therapeutic target to improve treatment responses. However, rather than viewing NF-κB modulation and microbiota-targeted therapies as mutually exclusive strategies, a synergistic approach integrating both avenues may offer greater therapeutic efficacy. This is particularly relevant given the bidirectional crosstalk between NF-κB and the gut microbiota, wherein dysbiosis can drive NF-κB activation, and NF-κB signalling in turn shapes microbial composition and gut barrier function. The gut microbiota, comprising trillions of microorganisms, plays an important role in systemic immune regulation and inflammation [9]. Certain commensal bacteria appear to modulate host immune responses in ways that enhance the efficacy of anticancer treatments like immune checkpoint inhibitors (ICIs) and chemotherapy. Dysbiosis, characterised by a loss of microbial diversity and an imbalance between beneficial and pathogenic taxa, is increasingly recognised as a contributor to chronic NF-κB activation [10, 11]. Disrupted gut permeability, commonly referred to as “leaky gut,” allows microbial products such as lipopolysaccharides (LPS) to translocate into systemic circulation, where they engage Toll-like receptor 4 (TLR4), triggering NF-κB activation and chronic inflammation [12, 13]. Such processes create a pro-inflammatory microenvironment conducive to cancer development and progression [14].

Cancer therapies exacerbate these challenges [15]. ICIs, while revolutionising oncology, frequently induce immune-related adverse events (irAEs), including colitis, by disrupting gut homeostasis and amplifying NF-κB signalling [16, 17]. Chemotherapeutic agents, such as irinotecan and 5-fluorouracil (5-FU), compromise gut epithelial integrity, enabling microbial translocation and fuelling systemic inflammation [18, 19]. Similarly, radiotherapy-induced dysbiosis can impair tumour radiosensitivity, negatively impacting treatment outcomes [20].

Mechanistically, the gut microbiota exerts its influence on NF-κB through metabolites such as short-chain fatty acids (SCFAs), secondary bile acids, and microbial structural components [21]. SCFAs, particularly butyrate, suppress NF-κB activation by inhibiting histone deacetylase (HDAC) activity and promoting IκBα resynthesis, thereby dampening inflammation and preserving epithelial barrier function [22, 23]. Recognising these intricate interconnections, gut modulation strategies such as dietary interventions, probiotics, prebiotics, and FMT have gained attention as therapeutic adjuncts in oncology [24]. By restoring microbial diversity and mitigating NF-κB-driven inflammation, these interventions may offer a means of enhancing the efficacy of cancer treatments while reducing adverse effects [24, 25]. For instance, clinical trials have demonstrated that FMT can improve ICI response rates by modulating immune activation pathways, including NF-κB [24, 26]. A summary of clinical studies exploring microbiota-targeted interventions in cancer therapy is presented in Table 1.

**Table 1:** Bacterial taxa frequently implicated in gastrointestinal malignancies and therapy response modulation

Microbial species	Mechanism of action	Cancer type	Impact on NF-κB / immune pathway
*Fusobacterium nucleatum*	Promotes tumour invasion, binds E-cadherin, activates β-catenin	Colorectal cancer	Activates NF-κB and inhibits T-cell function [27]
*Enterotoxigenic Bacteroides fragilis*	Secretes BFT toxin; disrupts tight junctions	Colorectal cancer	Induces IL-17, TNF-α, and NF-κB activation [28, 29]
*Helicobacter pylori*	Induces CagA-dependent inflammation	Gastric cancer	Stimulates NF-κB, IL-8 expression, and chronic inflammation [30, 31]
*Akkermansia muciniphila*	Improves barrier integrity; stimulates immune modulation	Multiple cancer types (adjunct in ICI)	Associated with improved ICI response; reduces inflammation [32]

These species influence host immunity and tumour progression through NF-κB-related inflammatory signalling and epithelial barrier disruption

This review explores the complex interplay between NF-κB signalling, gut microbiota, and cancer therapies. It synthesises current evidence on gut permeability, microbial metabolites, and inflammation, highlighting the translational potential of gut-targeted therapies in addressing critical gaps in cancer care. This review focuses on how gut modulation specifically influences NF-κB-driven oncogenesis and therapy resistance.

### The NF-κB pathway: mechanisms and regulation

**Fig. 1:**
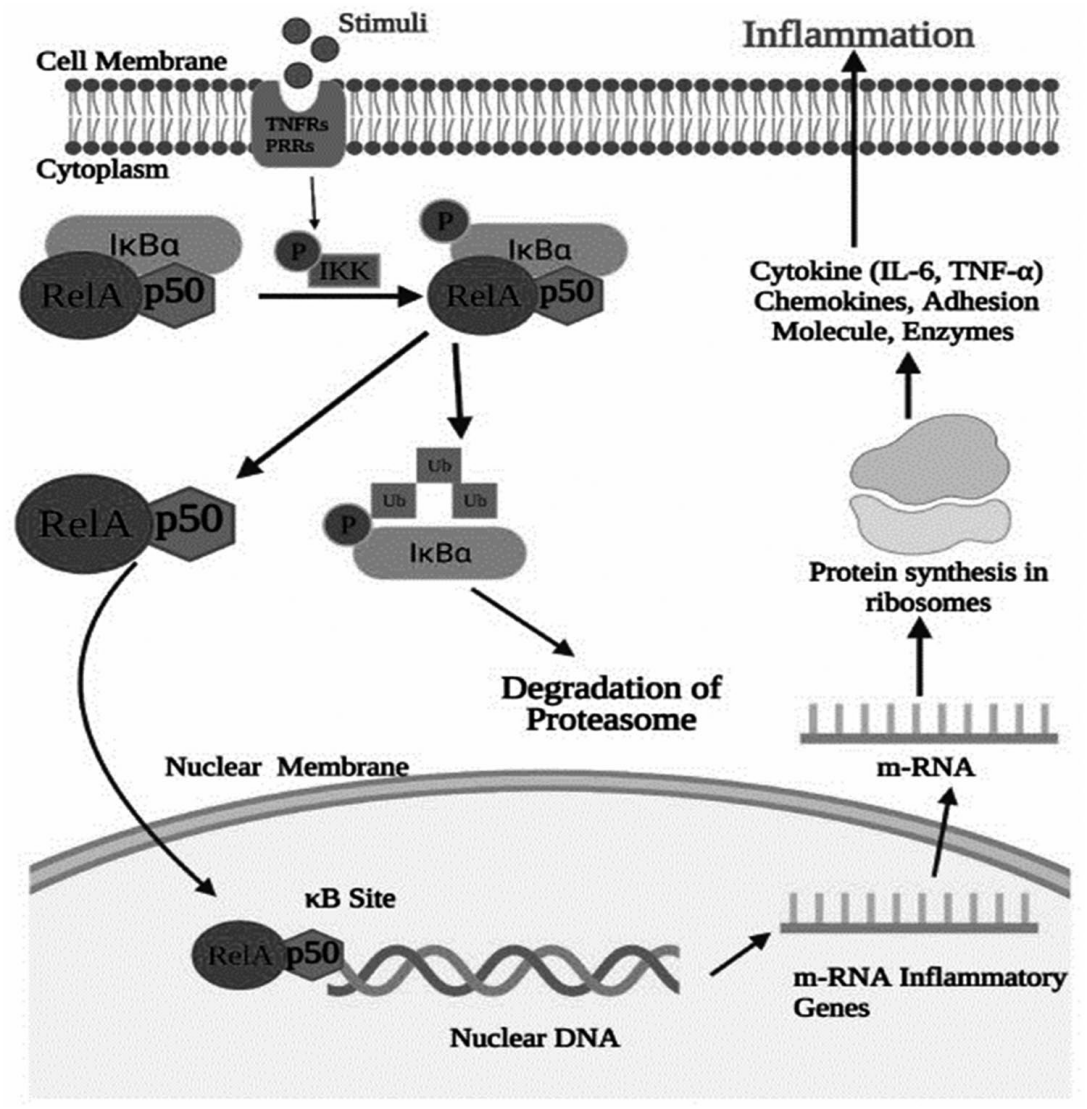
Upon stimulation by extracellular ligands binding to TNF receptors (TNFRs) or pattern recognition receptors (PRRs), the IκB kinase (IKK) complex is activated, leading to the phosphorylation and ubiquitination of IκBα. This process results in proteasomal degradation of IκBα, freeing the NF-κB dimer (RelA/p50) to translocate into the nucleus. Once in the nucleus, NF-κB binds to κB sites in the DNA, promoting the transcription of pro-inflammatory genes, including cytokines (e.g., IL-6, TNF-α), chemokines, adhesion molecules, and enzymes. These gene products drive inflammatory responses and protein synthesis in the ribosome, contributing to cellular and systemic inflammation [33]

The NF-κB signalling pathway plays a central role in cellular processes including immune responses, inflammation, apoptosis, and cellular proliferation (Fig. 1). This highly conserved pathway is tightly regulated through intricate molecular mechanisms that ensure appropriate cellular responses to stimuli [34]. The NF-κB family consists of five transcription factors: RelA (p65), RelB, c-Rel, NF-κB1 (p50/p105), and NF-κB2 (p52/p100) [35]. These proteins share a conserved Rel Homology Domain (RHD), which mediates DNA binding, dimerisation, and interaction with inhibitory proteins, while also enabling nuclear localisation (Kaltschmidt et al., 2022). Based on structural and functional differences, the family is divided into two groups: Rel proteins (RelA, RelB, and c-Rel), which possess transcription activation domains (TADs), and NF-κB1 and NF-κB2, which lack TADs in their mature forms (p50 and p52) and require co-activators such as Bcl-3 for transcriptional activity [6]. The precursor proteins, p105 and p100, undergo controlled proteasomal processing to generate their mature forms, p50 and p52 [35]. This process involves key structural domains, including the PEST domain and the glycine-rich region (GRR) (Kaltschmidt et al., 2022). The PEST domain undergoes phosphorylation, triggering ubiquitin-mediated processing, while the GRR halts proteasomal degradation at precise points to generate functional subunits (Kaltschmidt et al., 2022). This proteolytic mechanism exemplifies the sophisticated control underpinning NF-κB activation.

The versatility of NF-κB is reflected in its ability to form homodimers or heterodimers, which bind to κB enhancer sequences in the promoters of target genes, influencing diverse biological outcomes [37]. For example, p50 and p52 homodimers typically repress transcription unless paired with TAD-containing subunits like RelA [38]. Conversely, RelA-containing dimers robustly activate transcription in response to pro-inflammatory signals [6]. This dynamic regulation ensures cellular responses remain context-specific, balancing activation and repression to maintain homeostasis. The activity of NF-κB is further regulated by the IκB family of inhibitory proteins, which sequester NF-κB dimers in the cytoplasm (Zhang et al., 2021). These proteins prevent nuclear localisation by masking nuclear localisation signals (Kaltschmidt et al., 2022). Upon stimulation, inhibitory κB (IκB) proteins are phosphorylated by the IκB kinase (IKK) complex, ubiquitinated, and degraded via the 26S proteasome, freeing NF-κB dimers to translocate to the nucleus [14]. This cyclical activation and inhibition tightly control NF-κB signalling to prevent excessive or inappropriate responses, which could otherwise lead to pathological conditions such as chronic inflammation or cancer [7].

### Canonical pathway

**Fig. 2:**
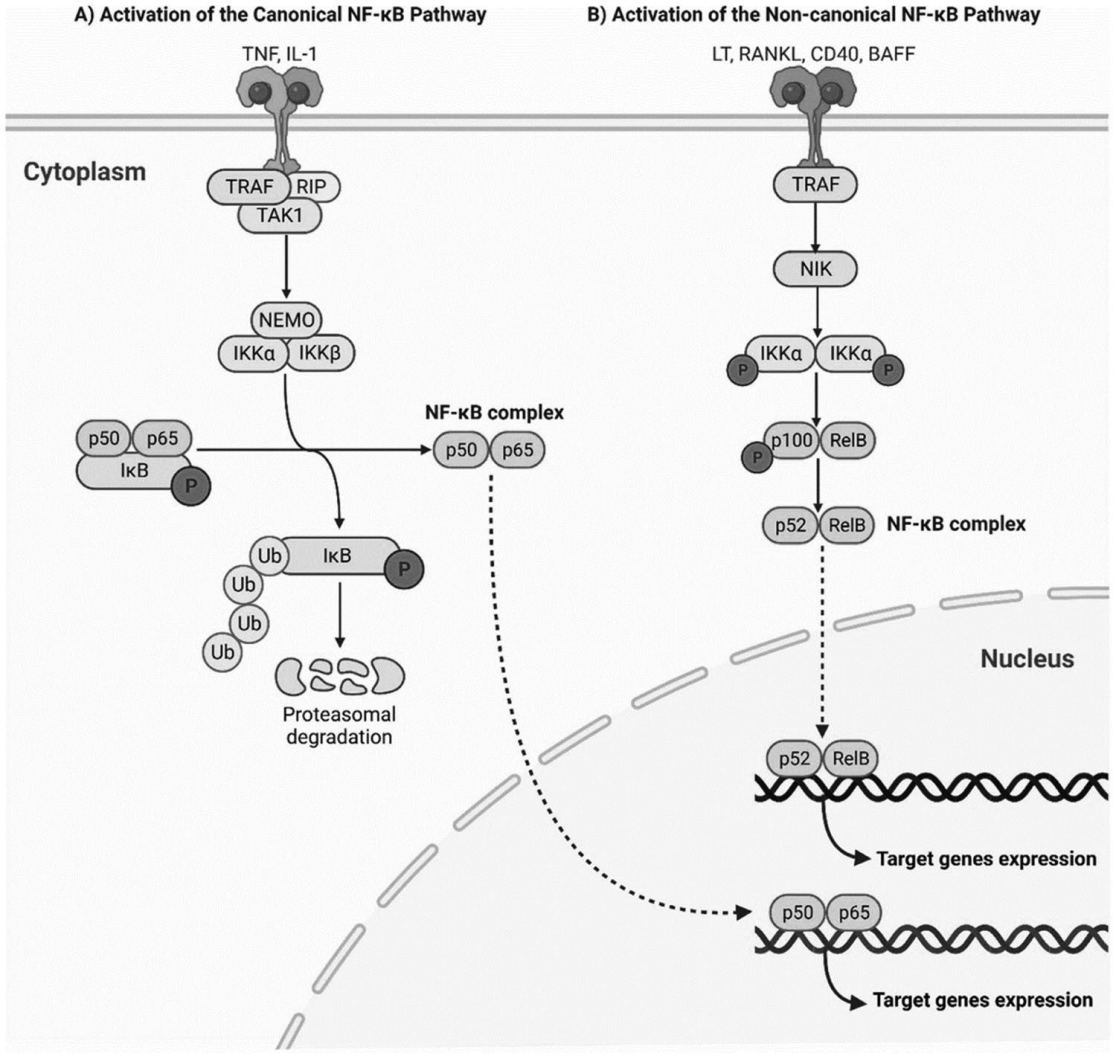
NF-κB Activation Pathways | Upon stimulation by diverse ligands, the IKK complex is activated, initiating the phosphorylation and ubiquitination of IκB proteins. This process leads to IκB degradation and the release of NF-κB dimers. Subsequently, NF-κB dimers undergo post-translational modifications that facilitate their nuclear translocation. Once in the nucleus, NF-κB binds to specific DNA sequences, driving the transcription of target genes involved in inflammation, immunity, and cell survival [39]

The canonical NF-κB pathway (Fig. 2) is the most extensively studied and is crucial in regulating innate immunity and inflammation [40]. This pathway is typically activated by pro-inflammatory stimuli such as TNF-α, interleukin-1 beta (IL-1β), and microbial components like LPS [41]. These stimuli activate cell surface receptors, including Toll-like receptors (TLRs) and TNF receptors (TNFRs), which recruit adaptor proteins such as MyD88 and TRIF to form signalling complexes [41]. The activation of these complexes leads to the phosphorylation and subsequent degradation of IκB proteins by the IKK complex, which comprises IKKα, IKKβ, and the regulatory subunit NEMO (IKKγ) [42]. This process releases p65/p50 dimers, which translocate to the nucleus to initiate the transcription of target genes involved in inflammation (e.g., TNF-α, IL-6) and cell survival (e.g., Bcl-2) [40]. The canonical pathway is tightly controlled by feedback loops, such as the resynthesis of IκBα, which binds nuclear NF-κB and shuttles it back to the cytoplasm [41]. Dysregulation of this pathway can lead to chronic inflammation and is implicated in various diseases, including cancer, where persistent NF-κB activation promotes tumourigenesis by facilitating epithelial-mesenchymal transition (EMT), angiogenesis, and immune evasion [43].

### Non-canonical pathway

In contrast, the non-canonical NF-κB pathway is activated by a narrower range of stimuli, including members of the TNF receptor superfamily such as lymphotoxin-β receptor (LTβR), B-cell activating factor (BAFF), and receptor activator of nuclear factor-kappa B ligand (RANKL) [44] (Fig. 2). This pathway relies on NF-κB-inducing kinase (NIK), which stabilises and activates IKKα independently of NEMO [45]. IKKα then phosphorylates the p100 precursor protein, leading to its partial proteasomal processing into p52. The resultant p52/RelB heterodimer translocates to the nucleus and regulates genes involved in adaptive immunity and lymphoid organogenesis [44]. Unlike the canonical pathway, the non-canonical pathway is characterised by delayed but sustained activation [46]. This slower response is tightly regulated to ensure specificity, with p100 acting as an inhibitory molecule in the absence of appropriate stimuli [41]. Emerging evidence suggests that microbial metabolites such as SCFAs influence the non-canonical pathway by modulating immune homeostasis [45]. For instance, butyrate enhances regulatory T-cell function and suppresses pro-inflammatory cytokines, indirectly supporting non-canonical NF-κB activity [47].

### Negative feedback loops and IκB dynamics

Central to NF-κB regulation are the IκB proteins, which form negative feedback loops to prevent sustained activation [48]. The primary IκB isoforms, IκBα, IκBβ, and IκBε, sequester NF-κB dimers in the cytoplasm by masking their nuclear localisation signals, thereby inhibiting transcriptional activity [34]. Upon pathway activation, IκB proteins are phosphorylated by the IKK complex, leading to their ubiquitination and subsequent proteasomal degradation [5]. This releases NF-κB dimers, enabling their nuclear translocation and transcriptional activation of target genes [42].

A key feedback mechanism involves the resynthesis of IκBα, encoded by the NF-κB target gene Nfkbia [39]. Newly synthesised IκBα translocates to the nucleus, displaces NF-κB dimers from DNA, and exports them to the cytoplasm, reinstating an inactive state [7]. This cyclical regulation allows rapid activation and deactivation of NF-κB signalling, preventing excessive inflammatory responses [6]. In contrast, IκBε exhibits delayed kinetics, providing sustained feedback to moderate prolonged NF-κB activity under specific conditions [34]. Meanwhile, IκBβ displays a distinct role by binding to RelA and c-Rel within the nucleus, counteracting IκBα-mediated inhibition and promoting the transcription of a subset of target genes [8]. These functional distinctions highlight the nuanced contributions of different IκB isoforms to NF-κB regulation.

## Interplay between canonical and non-canonical pathways

While the canonical and non-canonical pathways are distinct, they exhibit significant cross-regulation. Certain stimuli, such as BAFF and CD40L, can activate both pathways, enabling a coordinated response [41]. For example, canonical NF-κB activation can upregulate the expression of non-canonical components like NIK, bridging the two pathways [41]. Dysregulation of this interplay contributes to various diseases, including chronic inflammatory conditions and cancer [39]. Persistent activation of the canonical pathway in an inflammatory microenvironment promotes tumourigenesis through mechanisms such as angiogenesis, EMT, and immune evasion [7]. Simultaneously, aberrant activation of the non-canonical pathway can impair immune regulation and lymphoid organogenesis [36].

### Cancer: development and hallmarks

Cancer progression involves a sequential process of initiation, promotion, and progression, ultimately resulting in the acquisition of hallmark traits such as sustained proliferative signalling, immune evasion, angiogenesis, and metastatic potential [49]. These hallmarks, as conceptualised by Cavalleo and colleagues [50], arise from intricate interactions between genetic mutations, epigenetic modifications, environmental factors, and chronic inflammation. Dysregulation of NF-κB not only drives aberrant tumour cell behaviour but also alters the tumour microenvironment (TME), establishing it as a potential target for therapeutic intervention in cancer [6].

### Development of cancer stem cells and cancer origins

Cancer stem cells (CSCs) represent a sub-population within tumours defined by their ability to self-renew, differentiate, and drive tumour initiation, progression, and recurrence [36]. These cells are implicated in tumour heterogeneity, therapeutic resistance, and disease aggressiveness [51]. While early models suggested CSCs originate from normal stem cells, such as haematopoietic stem cells (HSCs), evidence now indicates that CSCs can also arise from differentiated cancer cells undergoing dedifferentiation under the influence of the TME [52] The dynamic interactions between CSCs and their surrounding stromal cells, immune infiltrates, and extracellular matrix components are mediated by pathways such as NF-κB, Wnt/β-catenin, and Hedgehog [53–55]. Notably, NF-κB activation promotes CSC survival, enhances multi-lineage differentiation, and promotes resistance to therapeutic interventions [36, 56]. For instance, pro-inflammatory cytokines within the TME, such TNF-α, activate NF-κB to upregulate anti-apoptotic genes and drive clonal expansion of CSCs [57–59]. These interactions highlight the importance of targeting NF-κB signalling within the CSC niche, particularly through gut modulation strategies aimed at disrupting these pro-tumorigenic pathways [36].

### Microenvironment and macroenvironment of cancers

The TME is a dynamic and heterogenous milieu comprising cancer cells, stromal cells, immune cells, and extracellular matrix [58]. Interactions within this environment sustain tumour growth, drive metastasis, and mediate immune evasion [21, 49]. NF-κB orchestrates these interactions by regulating cytokine production, angiogenic factors, and cell adhesion molecules [60]. For example, vascular endothelial growth factor (VEGF), regulated by NF-κB, promotes angiogenesis to ensure a sufficient nutrient and oxygen supply for tumour expansion [61]. Angiogenesis is essential for tumour growth and metastasis, providing a vascular network for nutrient supply and dissemination [62]. NF-κB regulates pro-angiogenic factors such as VEGF and angiopoietin-2, often in cooperation with hypoxia-inducible factor 1-alpha (HIF-1α) [63]. Hypoxic conditions within tumours amplify NF-κB activity, sustaining angiogenesis in a feedback loop [64]. Beyond the localised TME, systemic changes within the tumour macroenvironment include alterations in immune cell populations and metabolic pathways [61]. Tumour-associated macrophages (TAMs), often polarised to an immunosuppressive M2 phenotype by NF-κB signalling, secrete cytokines such as interleukin-10 (IL-10) and transforming growth factor-beta (TGF-β) [65]. These cytokines suppress cytotoxic T-cell activity, enabling tumour cells to evade immune surveillance [66]. Similarly, myeloid-derived suppressor cells (MDSCs) and regulatory T cells (Tregs) contribute to systemic immune suppression, creating a permissive environment for tumour progression and metastasis [67, 68].

### Cellular hierarchy and therapy resistance in CSCs

The hierarchical model of tumour organisation places CSCs at the apex, with progenitor-like cells and differentiated tumour cells arising from this sub-population [69]. CSCs exhibit unique attributes, including self-renewal, multi-lineage differentiation potential, and inherent resistance to therapy [70]. These properties are mediated by molecular markers such as CD44, CD133, and ALDH1, which are associated with poor clinical outcomes [51, 71]. Therapeutic resistance in CSCs arises from multiple mechanisms, including enhanced DNA repair capacity, immune checkpoint upregulation, and efflux pump activity [69]. NF-κB further exacerbates these resistance pathways by upregulating anti-apoptotic proteins such as Bcl-2 and survivin, and by activating transcriptional programs that sustain the CSC phenotype [72].

### Progression and metastasis: acquiring malignant traits

Progression and metastasis represent the culmination of molecular and cellular changes that enable tumour cells to invade surrounding tissues, enter the bloodstream, and colonise distant sites [67]. These processes are driven by hallmark features such as EMT, angiogenesis, immune evasion, and metastasis, with NF-κB at the centre of their regulation [73]. EMT is a biological process wherein epithelial cells acquire mesenchymal properties, losing polarity and adhesion while gaining motility and invasive capabilities [74]. NF-κB drives EMT by inducing transcription factors such as Snail, Twist, and Slug, which repress epithelial markers (e.g., E-cadherin) and upregulate mesenchymal markers (e.g., vimentin) [75]. Dysbiosis-associated bacteria such as *Fusobacterium nucleatum* exacerbate EMT by activating TLR4-NF-κB signalling, promoting tumour invasiveness, particularly in colorectal cancer [76, 77]. Pro-inflammatory cytokines within the TME, including TNF-α and IL-6, further increase NF-κB-driven EMT, linking inflammation to metastasis [78, 79].

### Immune evasion and metastatic niche formation

Immune evasion is a defining feature of cancer progression, enabling tumours to escape cytotoxic immune responses[80]. NF-κB supports this process by upregulating immune checkpoint molecules like PD-L1 and recruiting immunosuppressive cell populations, including TAMs, MDSCs, and Tregs [81]. These interactions suppress anti-tumour immunity and create an environment conducive to tumour growth [82]. Metastatic dissemination is further facilitated by NF-κB-regulated genes involved in cell adhesion, migration, and invasion [83].

### Gut microbiota and cancer development

The relationship between the gut microbiota and cancer is particularly evident in the development of gastric and colorectal malignancies [23, 25, 84]. *Helicobacter pylori* (*H. pylori*) is a well-documented driver of gastric cancer through its ability to induce chronic gastritis and β-catenin signalling [30]. Clinical studies have demonstrated that eradicating *H. pylori* reduces the risk of gastric cancer, highlighting the potential for microbial modulation in cancer prevention[31, 85, 86]. In colorectal cancer, microbial shifts towards pathogenic taxa such as *Bacteroides fragilis*, *Fusobacterium nucleatum*, and *Porphyromonas asaccharolytica* exacerbate NF-κB activation and inflammation [27, 28, 87, 88]. *Enterotoxigenic Bacteroides fragilis* (ETBF), for example, secretes a metalloprotease toxin that disrupts epithelial tight junctions and drives IL-17-mediated inflammation via NF-κB [89]. Preclinical models have further demonstrated that the gut microbiota influences tumour initiation and progression [90]. Germ-free mice colonised with faecal samples from colorectal patients develop tumours, while microbial depletion in genetically predisposed models suppresses lung cancer development [91]. Most clinical studies assessing the gut microbiota rely on faecal samples due to their non-invasive accessibility, enabling broad population-level comparisons of microbial composition. In contrast, preclinical models often utilise mucosal biopsies or caecal contents to capture spatial and mechanistic nuances in host–microbiota interactions [90, 91] (Fig. S1).

### Overview of cancer treatment modalities

The therapeutic landscape for colorectal and gastric cancers has evolved to embrace a multidisciplinary approach, integrating surgical resection, chemotherapy, radiotherapy, targeted therapies, and immunotherapy. Treatment selection is typically based on tumour stage, histopathological features, molecular profiling, and patient-specific factors. Surgical resection remains the cornerstone of curative intent in early-stage gastrointestinal cancers. Procedures include local excision or segmental colectomy for colorectal lesions, and subtotal or total gastrectomy with lymphadenectomy in gastric cancers. Perioperative strategies incorporating chemotherapy or chemoradiation are employed to reduce recurrence and enhance survival outcomes [92]. Chemotherapy plays a central role across stages—from neoadjuvant and adjuvant regimens to palliative care. Standard protocols include FOLFOX (5-fluorouracil, leucovorin, oxaliplatin) and FOLFIRI (5-fluorouracil, leucovorin, irinotecan) in colorectal cancer, while gastric cancer treatments often utilise cisplatin-based combinations such as ECF (epirubicin, cisplatin, 5-FU). While effective, these regimens are associated with epithelial toxicity, disruption of the intestinal barrier, and activation of pro-inflammatory pathways such as NF-κB, thereby potentiating adverse effects [92]. Radiotherapy, commonly used for rectal cancer and in certain gastric protocols, induces DNA damage and oxidative stress, which promote local cytokine release and inflammatory signalling. These changes can upregulate NF-κB and contribute to therapy resistance and tissue injury [93].

Targeted therapies have advanced precision oncology by disrupting tumour-specific pathways. In colorectal cancer, epidermal growth factor receptor (EGFR) inhibitors like cetuximab and panitumumab are prescribed in RAS wild-type tumours, whereas angiogenesis is suppressed using VEGF inhibitors such as bevacizumab. HER2-targeted agents (e.g., trastuzumab) are used in HER2-positive gastric adenocarcinoma. However, treatment responses can be compromised by tumour heterogeneity and inflammatory feedback loops that activate NF-κB signalling [94]. Immunotherapy has redefined treatment for mismatch repair-deficient (dMMR) or microsatellite instability-high (MSI-H) colorectal and gastric cancers. Immune checkpoint inhibitors (ICIs), including pembrolizumab and nivolumab, show significant efficacy in these immunogenic subtypes. However, the gut microbiome plays a pivotal role in modulating ICI responsiveness. Dysbiosis has been associated with reduced therapeutic efficacy, while microbial restoration strategies, such as faecal microbiota transplantation (FMT) and probiotic supplementation, have been shown to enhance immunotherapy outcomes [21].

### Therapy resistance and treatment-induced dysbiosis

Therapy resistance is a significant barrier to effective cancer treatment, with the NF-κB pathway involved in mediating resistance to conventional therapies, such as chemotherapy and radiotherapy [37, 95]. Cancer treatments not only activate NF-κB-dependent survival pathways but also disrupt the gut microbiota, creating an inflammatory milieu that exacerbates tumour progression and treatment failure [15, 96]. While NF-κB activation in response to chemotherapy or radiotherapy promotes the transcription of anti-apoptotic genes, this process is tightly linked to therapy-induced dysbiosis. Disruption of microbial homeostasis can amplify NF-κB signalling, suggesting that therapeutic resistance may be more effectively addressed by targeting both the inflammatory signalling cascade and the gut microbial environment [97, 98]. For example, chemotherapy-induced DNA damage activates NF-κB via the IKK complex, leading to the upregulation of survival proteins such as myeloid cell leukemia-1 (MCL-1) and inhibitors of apoptosis proteins (IAPs) [37, 99]. This response is further enhanced in CSCs, which exhibit heightened NF-κB activity [36]. CSCs leverage this pathway to maintain quiescence, evade apoptosis, and resist chemotherapeutic agents, acting as reservoirs for tumour recurrence and metastasis [56]. Additionally, radiotherapy exacerbates NF-κB activation by inducing oxidative stress and inflammatory cytokines, which perpetuate tumour survival and immune evasion [100].

### Gut dysbiosis and its role in therapy resistance

Cancer therapies frequently disrupt the gut microbiota, leading to dysbiosis that fuels systemic inflammation and NF-κB activation [101]. Gut dysbiosis, characterised by reduced microbial diversity and an imbalance of beneficial and pathogenic species, exacerbates NF-κB-driven inflammation [11, 48]. Dysbiotic microbiota, such as *Enterobacteriaceae* and *Fusobacterium nucleatum*, release pro-inflammatory metabolites like trimethylamine-N-oxide (TMAO) and microbial components such as LPS. LPS engages TLR4 on intestinal and immune cells, triggering a canonical NF-κB cascade that perpetuates inflammation and systemic immune activation [102, 103]. *Fusobacterium nucleatum* exemplifies the pro-inflammatory and oncogenic roles of dysbiosis [27]. This bacterium produces virulence factors such as FadA adhesin, which binds to epithelial cadherins and activates NF-κB, promoting inflammatory and tumourigenic signalling [104, 105]. Such dysbiotic conditions advance EMT, immune evasion, and tumour progression in colorectal cancer [27, 106].

### Microbial interactions in cancer therapy resistance

Emerging evidence suggests that the gut microbiota modulates cancer therapy efficacy [19, 23, 29]. For example, pancreatic ductal adenocarcinoma (PDAC) samples enriched with *Gammaproteobacteria* metabolise gemcitabine into an inactive form via cytidine deaminase, reducing chemotherapy effectiveness[107]. Additionally, microbiota-induced NF-κB activation supports survival pathways in CSCs and promotes immune evasion, further contributing to therapeutic resistance [51]. Cancer therapies, including chemotherapy and ICIs, often disrupt gut homeostasis, inducing dysbiosis and amplifying systemic inflammation[92, 108–110]. Chemotherapeutic agents such as 5-fluorouracil (5-FU) deplete SCFA-producing bacteria, weaken epithelial barriers, and enable microbial translocation, which activates NF-κB and intensifies inflammation[92, 111]. This therapeutic dysbiosis poses a significant challenge by exacerbating treatment-related side effects and reducing efficacy [108, 110]. Radiotherapy similarly disrupts the gut microbiota, favouring the overgrowth of pathogenic taxa such as *Enterobacteriaceae* [93]. These bacteria release LPS, which activate NF-κB through TLR4, driving chronic inflammation and systemic immune activation [112]. This feedback loop not only supports tumour survival but also diminishes the efficacy of immunotherapies [91].

### Towards gut-targeted strategies in cancer therapy

Recognising the intricate relationship between the gut microbiota and NF-κB, gut-targeted interventions are being explored to improve cancer therapy outcomes [111]. Strategies such as dietary modulation, probiotics, and FMT have shown potential in restoring gut homeostasis, suppressing NF-κB activation, and mitigating therapy-induced inflammation [26, 113, 114].

### Dietary interventions

Diets rich in fibre, polyphenols, and omega-3 fatty acids have demonstrated the capacity to reshape gut microbial composition, enhance SCFA production, and may reduce NF-κB-mediated inflammation [77, 114–116]. Dietary fibre serves as a critical substrate for gut microbial fermentation, yielding SCFAs such as butyrate, acetate, and propionate[117]. SCFAs have been reported to exert anti-inflammatory effects through several mechanisms, many of which intersect with NF-κB signalling. One key mechanism is the inhibition of histone deacetylases (HDACs), which can lead to increased acetylation of NF-κB p65/p50 subunits and promote the sequestration of NF-κB by IκBα, limiting its transcriptional activity. Butyrate and propionate have been shown to act as HDAC inhibitors, resulting in decreased expression of NF-κB-dependent pro-inflammatory cytokines. Evidence suggests that butyrate has the most pronounced effect, followed by propionate and acetate, though the relative impact may vary depending on cellular context [118]. SCFAs also interact with G-protein-coupled receptors (GPCRs), including GPR41, GPR43 (FFAR2), and GPR109A (HCAR2). These receptors have been implicated in modulating immune responses, with some studies indicating that SCFA engagement with GPR43 can lead to a reduction in NF-κB activation through β-arrestin-2 signalling [119]. Additionally, butyrate activation of GPR109A has been associated with the suppression of NF-κB-driven inflammatory responses in colonic macrophages and dendritic cells [120].

SCFAs contribute to intestinal barrier integrity, modulating immune responses, and suppressing NF-κB activity [47] (Fig. S2). Clinical studies have shown that high-fibre diets improve cancer therapy outcomes [115]. For instance, a study by Song and colleagues (2015) reported that fibre-enriched diets reduced systemic inflammation and improved survival rates in colorectal cancer patients undergoing treatment. Polyphenol-rich foods, including green tea, berries, and dark chocolate, are metabolised by gut microbiota into bioactive compounds that may also inhibit NF-κB activity[77, 116]. Polyphenols such as epigallocatechin gallate (EGCG) and resveratrol have been shown to reduce tumour angiogenesis, suppress inflammation, and potentiate chemotherapy effects [77]. Other studies have also demonstrated that resveratrol modulated the gut microbiota to reduce inflammatory cytokine production, linking polyphenols to improved cancer outcomes [121, 122]. Omega-3 fatty acids, found in fatty fish and flaxseeds, reduce the abundance of pro-inflammatory taxa and enhance the production of SCFAs, thereby suppressing NF-κB activity [123]. A study by Delpino and Figueiredo (2022) reported that omega-3 supplementation improved therapeutic responses in cancer patients by decreasing NF-κB activation and systemic inflammation.

Several clinical studies have examined the effects of SCFAs on systemic inflammation and immune responses. A 2022 systematic review and meta-analysis incorporating 29 human studies found that SCFA administration or dietary interventions that enhance SCFA production were associated with reductions in at least one biomarker of systemic inflammation. In studies focusing on inflammatory conditions, participants receiving SCFA supplementation or SCFA-enhancing diets exhibited lower circulating levels of C-reactive protein (CRP), IL-6, and TNF-α compared to controls. SCFAs have also been linked to adaptive immune regulation.

SCFAs have been investigated for their potential role in cancer prevention and adjunctive treatment. Epidemiological data suggest that diets rich in fermentable fibre, which promote SCFA production, are associated with a reduced risk of colorectal cancer. In experimental models, butyrate has been shown to induce apoptosis and cell-cycle arrest in cancer cells, partially through NF-κB inhibition[125]. Some clinical studies have reported that higher intratumoral butyrate levels correlate with lower inflammatory scores and improved immune cell infiltration in colorectal cancer patients [126]. However, direct interventional trials evaluating SCFA supplementation in cancer are still limited, and further research is required to determine the therapeutic implications.

## Enhancing SCFA production

Prebiotics such as inulin, fructooligosaccharides (FOS), and galactooligosaccharides (GOS) serve as substrates for SCFA-producing bacteria. These bacteria collectively restore epithelial barrier function by upregulating tight junction proteins, thereby mitigating microbial translocation and subsequent systemic inflammation [22, 47]. Preclinical studies support the role of prebiotics in mitigating therapy-induced side effects. Inulin supplementation, for example, increased *Akkermansia muciniphila* abundance and reduced tumour progression in immune checkpoint inhibitor models [127]. Additionally, GOS has been shown to alleviate chemotherapy-induced mucositis by promoting microbial diversity and reducing intestinal inflammation [128]. Clinical trials further substantiate prebiotics’ potential. For instance, a study examining FOS supplementation in breast cancer patients undergoing chemotherapy reported improved gut microbial diversity and reduced systemic inflammation [129].

### Probiotics and cancer

Probiotics, defined as live microorganisms that confer health benefits when administered in adequate amounts, have gained increasing attention for their role in cancer modulation. Probiotics exert their effects through several mechanisms, including modulation of gut microbiota composition, enhancement of gut barrier integrity, and regulation of immune signalling pathways. Notably, specific probiotic strains have been shown to regulate the NF-κB pathway, a key driver of inflammation and tumorigenesis [130]. Probiotics such as *Lactobacillus rhamnosus*, *Bifidobacterium bifidum*, and *Akkermansia muciniphila* have demonstrated the ability to reduce NF-κB activation, suppressing the expression of pro-inflammatory cytokines such as TNF-α, IL-6, and IL-1β, which are involved in tumour progression [131].

A growing body of research highlights the potential role of *Lactobacillus casei* in supporting cancer treatment. Experimental studies have demonstrated that *L. casei* ATCC 393 exerts significant anti-proliferative and pro-apoptotic effects on colon carcinoma cells, both in vitro and in vivo. Administration of live *L. casei* led to a substantial reduction in tumour volume, attributed to the upregulation of TRAIL and the downregulation of Survivin, key regulators of apoptosis[132]. Similarly, a clinical study by Son & Cho [133] reported that *Bifidobacterium longum* enhanced the efficacy of immune checkpoint inhibitors (ICIs) by promoting CD8 + T-cell infiltration into tumours, leading to improved responses to anti-PD-L1 therapy.

Beyond alleviating treatment-related side effects, probiotics have been implicated in modulating the tumour microenvironment. *Akkermansia muciniphila*, a prominent gut bacterium, has been associated with positive systemic effects on host metabolism and favourable outcomes to checkpoint blockade in cancer immunotherapy. Recent research has identified a specific phospholipid from *A. muciniphila’s* cell membrane that induces homeostatic immune responses through a non-canonical TLR2–TLR1 signalling pathway. These findings suggest a mechanistic link between the modulation of the gut microbiota by *A. muciniphila* and enhanced efficacy of ICI [32]. Another randomised study in patients undergoing colorectal cancer treatment found that probiotic supplementation led to reductions in systemic inflammation markers such as CRP and IL-6, supporting the hypothesis that probiotics mitigate inflammation-mediated cancer progression[134].

Preclinical studies further support the role of probiotics in cancer therapy. In murine models, supplementation with *Bifidobacterium* breve resulted in a significant reduction in tumour growth and prolonged survival, attributed to enhanced immune activation and reduced NF-κB-mediated inflammation [135]. Similarly, *Lactiplantibacillus plantarum* has been shown to exert anti-proliferative effects on colorectal cancer cells by modulating gut microbiota-derived metabolites and inflammatory pathways[136]. A recent study demonstrated that probiotic administration led to an increase in beneficial SCFA-producing bacteria, enhancing the anti-tumour immune response by promoting regulatory T-cell differentiation and reducing myeloid-derived suppressor cells (MDSCs) that contribute to immune evasion[130].

### Faecal microbiota transplantation (FMT) and cancer

Faecal microbiota transplantation (FMT) has emerged as a novel approach for modulating the gut microbiome and restoring microbial diversity, particularly in cancer patients experiencing dysbiosis due to disease progression or treatment-induced microbiota alterations. By transferring stool from healthy donors to cancer patients, FMT aims to enhance immune function, improve treatment responses, and mitigate adverse effects associated with immunotherapy and chemotherapy[137].

FMT exerts its effects through the restoration of microbial diversity, enhancement of SCFA production, and modulation of immune checkpoint pathways. Dysbiosis has been implicated in reduced efficacy of ICIs, and FMT has been proposed as a strategy to reintroduce beneficial microbial species that can enhance anti-tumour immune responses [91]. FMT also influences gut permeability and systemic inflammation by increasing levels of beneficial bacteria such as *Akkermansia muciniphila* and *Faecalibacterium prausnitzii*, which have been linked to improved responses to cancer immunotherapies [138].

Recent clinical trials have explored the potential of FMT in improving cancer treatment outcomes. A landmark study in advanced melanoma patients demonstrated that FMT from immunotherapy-responsive donors enhanced the efficacy of PD-1 blockade therapy, with patients exhibiting increased tumour response rates and improved overall survival [138]. FMT has also shown promise in mitigating chemotherapy-induced toxicity. A study investigating FMT in colorectal cancer patients undergoing chemotherapy found that FMT administration reduced gastrointestinal toxicity, improved gut microbial composition, and enhanced treatment tolerance [139]. These findings highlight the potential of FMT as an adjunctive therapy to alleviate treatment-related complications. Preclinical evidence supports the role of FMT in modulating tumour growth and immune responses. In murine models of colorectal cancer, FMT from healthy donors resulted in reduced tumour burden, enhanced regulatory T-cell differentiation, and increased expression of anti-inflammatory cytokines [140].

While FMT presents a promising approach to enhancing cancer treatment outcomes, several challenges remain. The variability in donor microbiota composition, the risk of transferring pathogenic microbes, and the lack of standardised protocols pose barriers to widespread clinical implementation. Additionally, long-term safety data on FMT in cancer patients are still limited, necessitating further investigation into optimal donor selection, treatment frequency, and microbiota-targeted interventions.

## Challenges

Despite the promising advances in gut modulation strategies for cancer therapy, several challenges must be addressed before these approaches can be fully integrated into clinical practice. One of the foremost barriers to the widespread adoption of microbiota-targeted therapies is the lack of standardisation in microbiome profiling methods[141]. Variability in sequencing platforms, bioinformatics pipelines, and data processing techniques creates inconsistencies that impede reproducibility and cross-study comparisons [142]. These inconsistencies make it difficult to establish strong correlations between specific microbial signatures and cancer outcomes [91]. Standardised methodologies are needed for metagenomic sequencing, microbial culture techniques, and functional microbiome analysis to enable reliable integration into clinical practice [143].

Regulatory challenges also present significant barriers to the clinical adoption of microbiota-based therapies. FMT, probiotics, and prebiotics exist in a regulatory grey area, as they straddle classifications between dietary supplements and therapeutic agents [144]. While probiotics and prebiotics are generally considered safe under existing food regulations, their therapeutic use in oncology necessitates stricter oversight to ensure efficacy and safety [145]. FMT, in particular, faces substantial regulatory scrutiny due to concerns about pathogen transmission and long-term safety[145]. Donor selection remains a critical factor in ensuring safe FMT outcomes, as the introduction of undesirable microbial species could exacerbate cancer progression or trigger adverse immune reactions [146]. Rigorous screening protocols must include microbiological, genetic, and functional assessments to minimise risks [147]. Additionally, there is concern about the transfer of antibiotic resistance genes and the risk of horizontal gene transfer, which could have unpredictable consequences for cancer patients receiving FMT [147].

## Future research directions

The field of gut modulation in cancer therapy is rapidly evolving, but several research gaps remain. Longitudinal studies are needed to assess the durability of gut microbiota changes induced by interventions such as FMT, probiotics, and prebiotics [25]. These studies should also evaluate the long-term safety and efficacy of these therapies, particularly in the context of ICI and emerging treatments [148]. Establishing clear biomarkers for therapeutic response and understanding how microbial shifts correlate with clinical outcomes will be essential for advancing microbiota-targeted interventions [91]. Another critical area of exploration is combinatorial approaches. Combining probiotics with dietary interventions or prebiotics may enhance microbial diversity and increase SCFA production, thereby amplifying therapeutic benefits [149]. Clinical trials examining these synergistic strategies are essential to optimise gut modulation therapies for cancer patients [23, 47].

Next-generation microbiota-based interventions such as engineered bacterial therapeutics, and microbial consortia offer novel approaches to enhancing treatment efficacy [150]. These approaches aim to harness synthetic biology to design probiotic strains that can deliver targeted immunotherapies, metabolite production, or tumour-suppressing functions [145]. However, further preclinical and clinical studies are needed to validate their safety and effectiveness before they can be integrated into standard oncological care [151].

## Conclusion

The integration of gut-targeted therapies into oncology represents a promising strategy for improving cancer treatment outcomes. By modulating the gut microbiota, these interventions offer potential benefits in counteracting therapy-induced dysbiosis, reducing inflammation, and enhancing therapeutic efficacy across multiple cancer types. Emerging evidence from clinical and preclinical studies supports the use of probiotics, prebiotics, and FMT as adjuncts to existing treatment modalities, including ICIs and chemotherapy. FMT and probiotic-based interventions have shown potential in mitigating chemotherapy-induced gastrointestinal toxicity, reinforcing gut barrier integrity, and augmenting anti-tumour immune responses. Beyond their direct therapeutic effects, microbiota modulation may also contribute to reduced immune evasion and tumour progression, positioning gut-targeted approaches as a critical component of precision oncology. However, the clinical translation of these strategies requires addressing key challenges, including standardisation of treatment protocols, regulatory considerations, ethical implications, and long-term safety evaluation.

## Supplementary Information

Below is the link to the electronic supplementary material.

Supplementary file1 (PDF 3198 kb)

## Data Availability

No datasets were generated or analysed during the current study.

## Abbreviations

BAFF: B-cell activating factor
CD40L: CD40 ligand
CSC: Cancer stem cell
EMT: Epithelial-to-mesenchymal transition
FMT: Faecal microbiota transplantation
FOS: Fructooligosaccharides
GOS: Galactooligosaccharides
GRR: Glycine-rich region
HDAC: Histone deacetylase
HIF-1α: Hypoxia-inducible factor 1-alpha
ICI: Immune checkpoint inhibitor
IKK: IκB kinase
IL-6: Interleukin-6
IκB: Inhibitor of kappa B
LPS: Lipopolysaccharides
MCL-1: Myeloid cell leukemia-1
MDSCs: Myeloid-derived suppressor cells
MyD88: Myeloid differentiation primary response 88
NDCs: Non-digestible carbohydrates
NF-κB: Nuclear factor-kappa B
NIK: NF-κB-inducing kinase
PD-L1: Programmed death-ligand 1
PEST: Proline, glutamic acid, serine, and threonine domain
RANKL: Receptor activator of nuclear factor-kappa B ligand
SCFAs: Short-chain fatty acids
TAMs: Tumour-associated macrophages
TLR4: Toll-like receptor 4
TME: Tumour microenvironment
TNF-α: Tumour necrosis factor-alpha
Tregs: Regulatory T cells
TRIF: TIR-domain-containing adapter-inducing interferon-β
VEGF: Vascular endothelial growth factor
