# Supplementary material for: Gut modulation to regulate NF-κB in colorectal and gastric cancer therapy and inflammation
Source: Cancer Immunol Immunother. 2025 Jul 12;74(8):264. doi: 10.1007/s00262-025-04118-9 (PMC12255603; doi:10.1007/s00262-025-04118-9)
Supplement: Supplementary file 1 — Supplementary file1 (PDF 3198 kb) [file 262_2025_4118_MOESM1_ESM.docx]

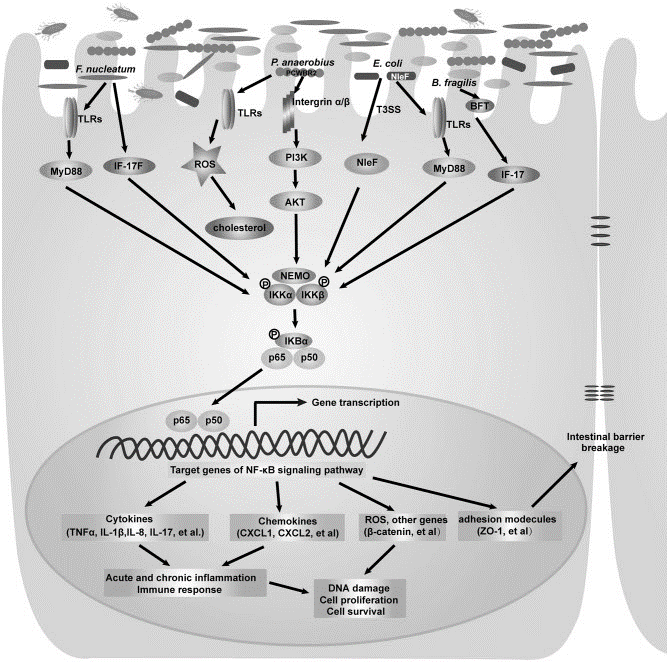


Figure S1. This illustration highlights the role of intestinal bacterial pathogens, including Fusobacterium nucleatum, Peptostreptococcus anaerobius, Escherichia coli, and Bacteroides fragilis, in activating the NF-κB signalling pathway in intestinal epithelial cells. These bacteria engage pattern recognition receptors such as TLRs and integrins to trigger downstream signalling cascades. Activation of the NF-κB pathway leads to the transcription of genes encoding pro-inflammatory cytokines, chemokines, adhesion molecules, and other factors. These gene products drive inflammation, DNA damage, cellular proliferation, and survival, while disrupting epithelial integrity [21].

**
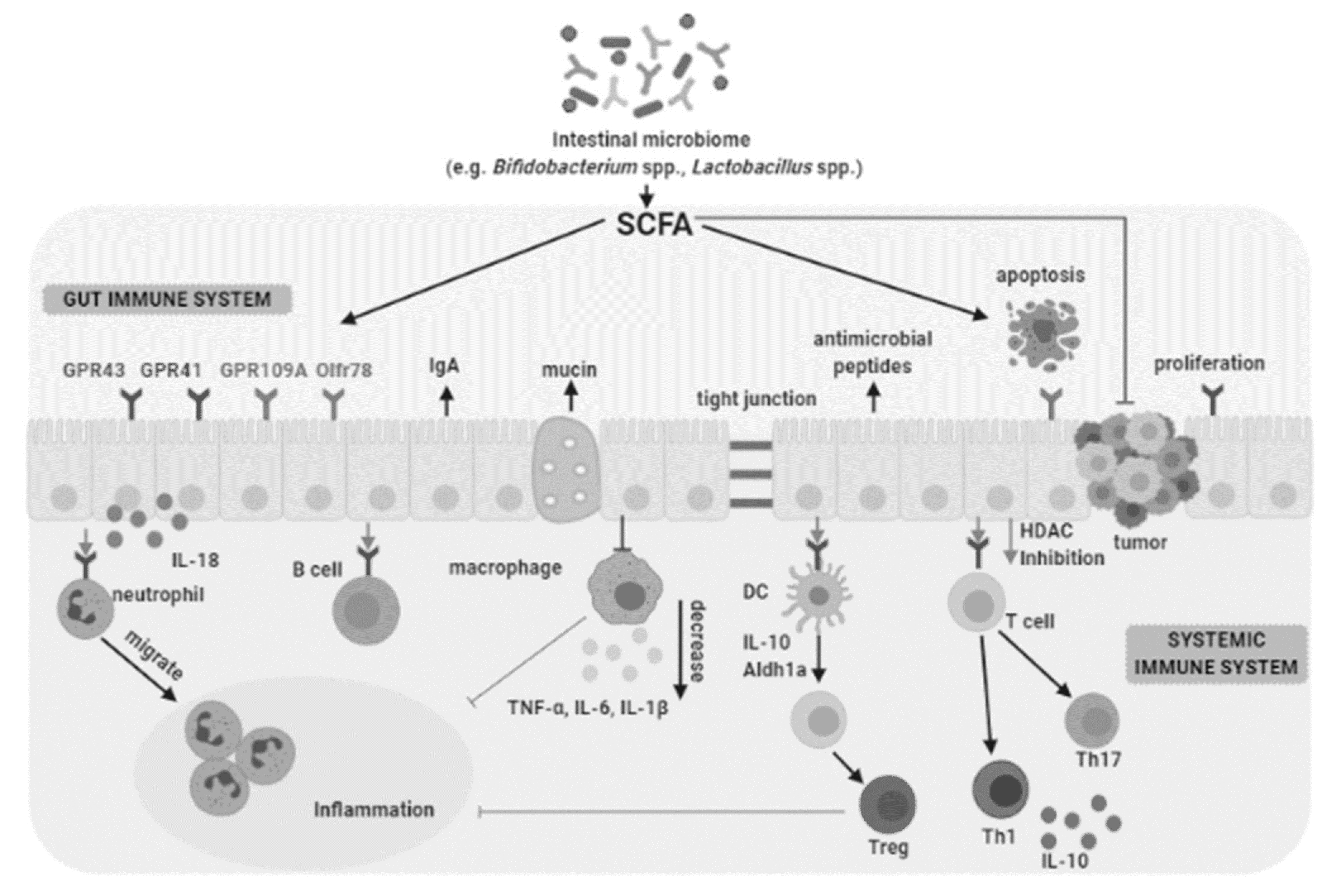
**

Figure S2. This figure illustrates the multifaceted roles of short-chain fatty acids (SCFAs), produced through the fermentation of non-digestible carbohydrates (NDCs) by gut microbiota, in regulating intestinal and systemic immune responses. SCFAs enhance intestinal barrier function by stimulating the secretion of interleukin-18 (IL-18), mucin (MUC2), and antimicrobial peptides while upregulating tight junction proteins. They modulate immune cell function via G-protein-coupled receptors (GPR41, GPR43, GPR109A), Olfr78 receptor signalling, and histone deacetylase (HDAC) inhibition, impacting nuclear factor-κB (NF-κB) activity. SCFAs facilitate neutrophil migration and phagocytosis, reduce macrophage-derived pro-inflammatory cytokines (e.g., IL-6, IL-1β, TNF-α), and enhance IgA production by B cells. Additionally, SCFAs influence T cell differentiation, promoting regulatory T cells (Tregs) and interleukin-10 (IL-10) production while suppressing tumour proliferation and promoting apoptosis, thereby reducing carcinogenesis. Abbreviations: Aldh1A2—aldehyde dehydrogenase 1A2. Arrows denote activation (→) and inhibition (⟞) [22].
